# Effects of intravenous hydration on risk of contrast induced nephropathy and in-hospital mortality in STEMI patients undergoing primary percutaneous coronary intervention: a systematic review and meta-analysis of randomized controlled trials

**DOI:** 10.1186/s12872-019-1054-y

**Published:** 2019-04-08

**Authors:** Yong Liu, Daqing Hong, Amanda Ying Wang, Rui Guo, Brendan Smyth, Jin Liu, Guoli Sun, Shiqun Chen, Ning Tan, Meg Jardine, David Brieger, Ahmed Shaman, Shariful Islam, Jiyan Chen, Martin Gallagher

**Affiliations:** 1 Department of Cardiology, Guangdong provincial Key Laboratory of Coronary Heart Disease Prevention, Guangdong Cardiovascular Institute, Guangdong General Hospital, Guangdong Academy of Medical Sciences, School of Medicine, South China University of Technology Guangzhou, Guangzhou, Guangdong China; 2 School of Medicine, South China University of Technology, Guangzhou, Guangdong China; 3 The George Institute for Global Health, the University of Sydney, Camperdown, Australia; 4 Renal Department, Sichuan Academy of Medical Science & Sichuan Provincial People’s Hospital, Chengdu, China; 5 Department of Neurosurgery, West China Hospital, Sichuan University, Chengdu, China; 6 King Saud University, Riyadh, Saudi Arabia; 7 Concord Clinical School, Sydney Medical School, University of Sydney, Camperdown, Australia

**Keywords:** Intravenous hydration, Contrast-induced nephropathy, ST-segment elevation-myocardial infarction, Primary percutaneous coronary intervention, acute kidney injury, dialysis, mortality

## Abstract

**Background:**

The role of intravenous hydration at the time of primary percutaneous intervention (PCI) for ST-segment elevation myocardial infarction (STEMI) remains unclear. Guidelines are vague, supported by low level evidence, and hydration is used less often than other clinical settings.To perform a systematic review and meta-analysis of all randomized controlled trials assessing intravenous hydration compared with non-hydration for prevention of contrast induced nephropathy (CIN) and In-hospital mortality in patients with STEMI undergoing primary PCI.

**Methods:**

Medline, EMBASE and the Cochrane Register were searched to September 2018. Included studies reported the incidence of CIN, In-hospital mortality, requirement for dialysis and heart failure. Relative risks with 95% confidence intervals (CIs) for individual trials were pooled using a random effects model.

**Results:**

Three moderate quality trials were identified including 1074 patients. Overall, compared with no hydration, intravenous hydration significantly reduced the incidence of CIN by 42% (RR 0.58; 95% CI: 0.45 to 0.74, *p* < 0.001). The estimated effects upon all-cause mortality (RR 0.56; 95% CI: 0.30 to 1.02, *p* = 0.057) and the requirement for dialysis (RR 0.52, 95% CI 0.14–1.88, *p* = 0.462) were not statistically significant. The outcome of heart failure was not consistently reported.

**Conclusions:**

Intravenous hydration likely reduces the incidence of CIN in patients with STEMI undergoing primary PCI. However, for key clinical outcomes such as mortality, heart failure and dialysis the effect estimates were imprecise. Further high quality studies are needed to clarify the appropriate volume of fluid and effects on outcomes.

**Electronic supplementary material:**

The online version of this article (10.1186/s12872-019-1054-y) contains supplementary material, which is available to authorized users.

## Background

Patients undergoing percutaneous coronary interventions (PCI) have a higher risk for the development of contrast induced nephropathy (CIN) [1]. CIN is associated with poorer outcomes for patients, including prolongation of hospital stay and higher mortality [2]. The risk in patients with ST-segment elevation-myocardial infarction (STEMI) undergoing primary PCI is even greater [3], and there are no treatments proven to mitigate this risk.

One recent study using intravenous hydration with normal saline before radiocontrast exposure, the cornerstone of CIN prevention across clinical medicine, showed a renal benefit for patients with STEMI [4]. But such therapy is less commonly used in patients undergoing primary PCI compared to elective PCI [5], probably due to the urgency of the procedure and concern about the development of congestive heart failure [6]. Clinical guidelines for PCI management recommend ‘adequate preparatory hydration’ [7], supported by lower level evidence, and more recent STEMI guidelines do not contain graded recommendations regarding the use of prophylactic hydration [8].

We performed a systematic review and meta-analysis of randomized controlled trials (RCT) in patients with STEMI undergoing primary PCI to examine the effect of prophylactic intravenous hydration compared to controls not receiving such hydration. The outcomes of CIN, requirement for dialysis, and In-hospital mortality were assessed.

## Methods

The research question, search strategy, inclusion criteria, and statistical analyses were pre-specified. All RCT assessing intravenous isotonic fluid hydration compared with non-hydration for prevention of CIN in STEMI patients undergoing primary PCI were included. No language or publication status restrictions were imposed. Participants of any age undergoing primary PCI were considered, as were studies utilising pre- or post-hydration strategies. Studies involving the co-administration of potential nephroprotective agents (eg. N-acetylcysteine) were excluded. The primary meta-analysis outcome was incidence of CIN, defined as an absolute increase in serum creatinine of ≥0.5 mg/dl (44 mmol/l) or a relative increase of ≥25% from the baseline value after administration of contrast media during primary PCI [4, 5, 9]. The secondary outcomes included all-cause In-hospital mortality (as reported by the studies) and CIN requiring dialysis. Heart failure or acute pulmonary edema events were also recorded using study author definitions.

### Search strategy and data collection

We searched MEDLINE, EMBASE, and Cochrane Central databases from the date of inception until September 2018. Tangential electronic exploration of related articles based on reference lists was also performed. Extensive hand searches of bibliographies of relevant reviews and related journals were also performed. Search terms included variants of hydration, fluid, nephropathy, contrast nephropathy, contrast-induced nephropathy, contrast media, contrast agent, kidney, renal, and myocardial infarction using text words and MeSH terms (see Additional file 1). Eligibility assessment of title and abstract and subsequent data extraction were performed independently by two authors (YH and DH) in an unblinded standardized manner using a study eligibility and data extraction form based on the Cochrane consumers and communication review group’s template. Conference abstracts and letters retrieved from electronic databases were also included in screening process. Any discrepancy was resolved by the third person (RG).

The quality of the studies was assessed according to Cochrane Handbook for Systematic Reviews of Interventions and GRADE.

### Statistical analysis

To assess study quality, we followed the guidelines in the Cochrane Handbook for Systematic Reviews of Interventions [10]. The primary outcome measure was quantified by computing the pooled risk ratio (RR) with 95% confidence interval (CI) using a random-effects model. Trial sequential analysis (TSA) was used to estimate the number of additional patients and events that would be required to demonstrate an effect of the intervention where non-significant effects currently exist. These values and cumulative Z curve were calculated, applying the O’Brien-Fleming monitoring boundaries, by Trial Sequential Analysis 0.9 Beta (Copenhagen Trial Unit, Copenhagen, Denmark) software [11]. To explore variability in study results we performed subgroup analysis comparing studies using isotonic saline versus those using sodium bicarbonate solutions. Heterogeneity was assessed using I^2^ statistic (with 95% CI) [12] where I^2^ values of 25, 50, and 75% may be considered as low, moderate, and high heterogeneity, respectively. The possibility of publication bias was assessed by a funnel plot and Egger’s regression asymmetry test [13]. Statistical analysis was performed using STATA software (version 13.0).

## Results

### Study selection

**Fig. 1:**
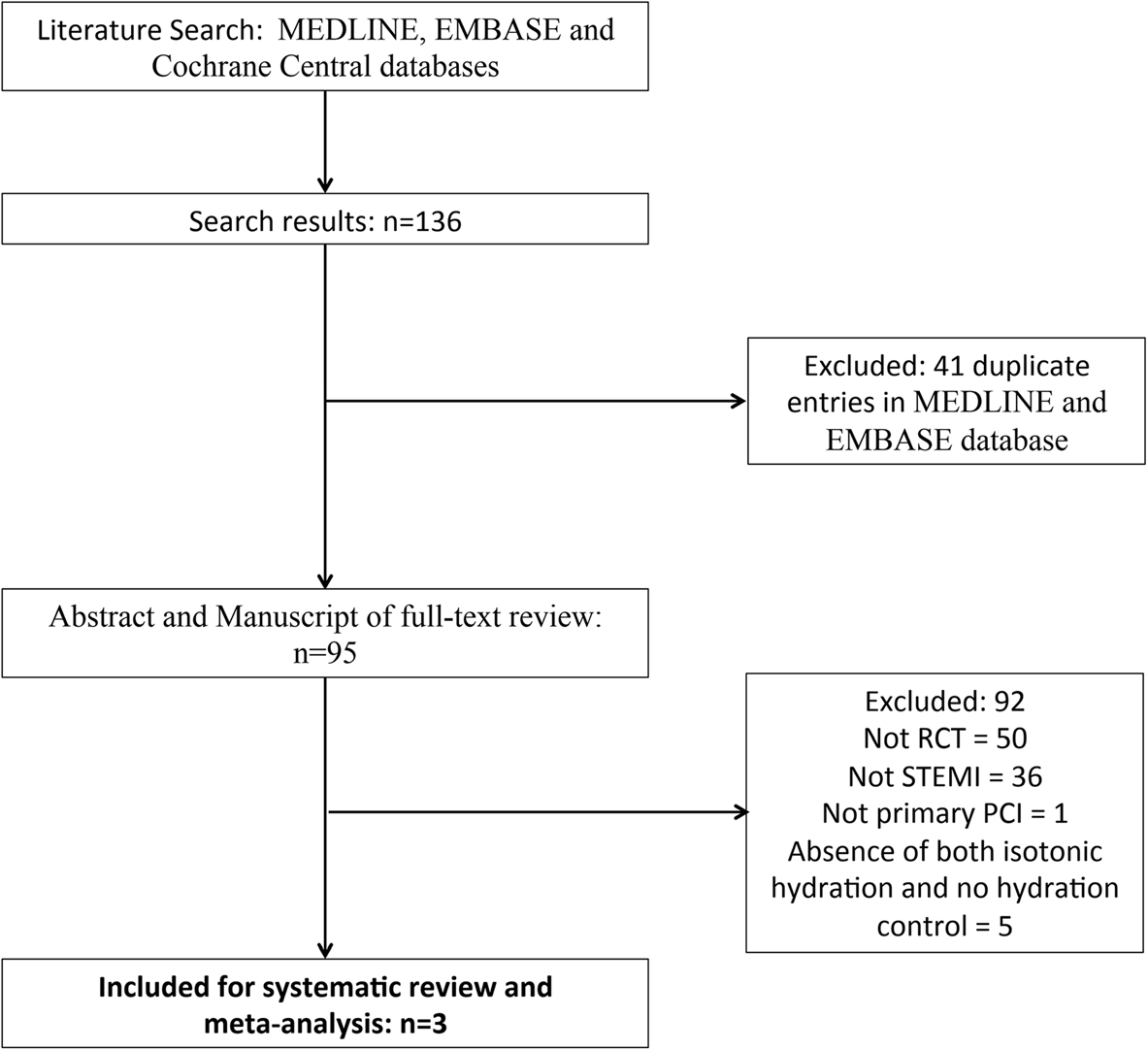
Study flow diagram of study selection

The search yielded a total of 143 citations of which 99 were selected for full-text review. A flow diagram of study selection is presented in Fig. 1.

### Study characteristics

Three RCTs were selected for the review and meta-analysis all of which reported the incidence of CIN as defined above. All three studies were single centre studies that enrolled adult patients with STEMI undergoing primary PCI. A total of 1085 patients were randomized, of whom 1074 were included in the primary analysis. Individual study characteristics, patient populations and key outcomes are presented in Table 1 and Additional file 2: Table S1.

**Table 1:** Baseline characteristics of included studies

Study	Country, recruitment period	No. of patients randomized	Age ±SD/ (range)	Killip class > 1	eGFR	Intervention Arm	Delivered hydration in intervention group^a^	CIN (%)	RRT(%)	In-hospital mortality (%)	HF /APE	Mean LOS (intervention; control) (days)
Maioli et.al.(2011)	Italy, July 2004–December 2008	461	65.0 ± 12.34	20.6% (93/450)	75.67 ± 21.71	Biocarbonate Solution: 3 mL/kg in 1 h, starting in the DR, 1 mL/kg per hour for 12 h after PCI, but reduced to 0.5 mL/kg in patients with LVEF≤40% or NYHA class III-IV Normal Saline: 1 mL/kg per hour for 12 h immediately after PCI, but reduced to 0.5 mL/kg in patients with LVEF≤40% or NYHA class III-IV	Mean volume: 1021 ± 196 ml	20.6%(93/450)	0.9% (4/450)	3.5% (16/450)	Unknown	Unknown
Luo et.al.(2014)	China, August 2009–October 2012	216	67.0 (57–75)	35.6% (77/216)	70.55 ± 22.90	Normal Saline: 1 mL/kg per hour for 12 h after primary PCI, but reduced to 0.5 mL/kg.hr. in patients with LVEF≤30% or Killip class II-III	Mean rate: 0.75 ml/kg/hour	27.8% (60/216)	3.7% (8/216)	6% (13/216)	6% (13/216)	6.8 ± 25.7; 14.4 ± 30.5
Jurado-Román et.al.(2015)	Spain, July 2012–November 2013	408	62.8 ± 13.04	14.7% (60/408)	89 ± 40.97	Normal Saline: 1 mL/kg/h since the beginning of procedure and during the following 24 h, but reduced to 0.5 mL/kg in patients with LVEF< 40% or Killip class III-IV	Mean volume: 1720 ± 234 ml	14%(57/408)	1% (3/303)	4.81%(15/312)	Unknown	6; 8.2

*ER* emergency room, *RRT* Requirement for dialysis, *HF* heart failure, *APE* Acute pulmonary edema, *LOS* Length of stay. Other abbreviations as in study design

^a^No paper reported hydration volume or rate in the control group

### Risk of bias and quality of clinical trials

**Fig. 2:**
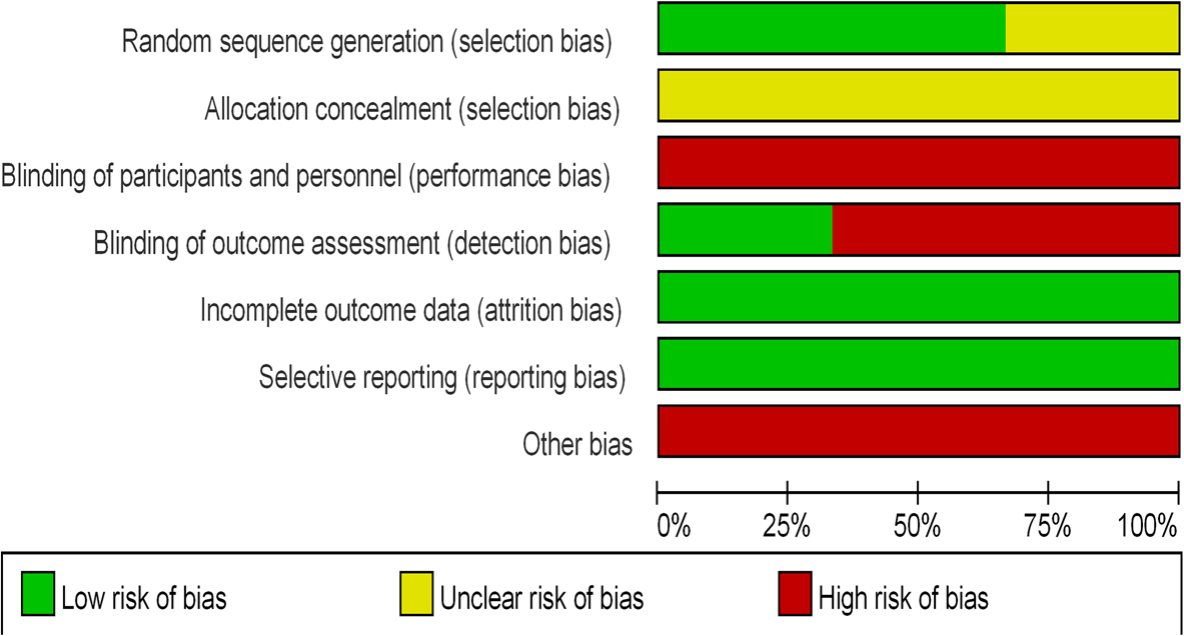
Risk of bias graph: ach risk of bias item presented as percentages across all included studies

The individual studies were judged to be of moderate quality with the risk of bias is summarised in Fig. 2 and Additional file 3: Figure S1. In addition to the absence of allocation concealment and blinding, other potential indicators of moderate study quality were that no study reported the volume of intravenous fluid received by the control group and that reporting of treatment variation (eg. reduction in infusion rate or cessation of hydration) in response to the development of pulmonary edema or hypotension was inconsistent. GRADE tool also suggested high quality for quality of evidence at CIN and in-hospital mortality (Additional file 4: Figure S2).

### Incidence of CIN

**Fig. 3:**
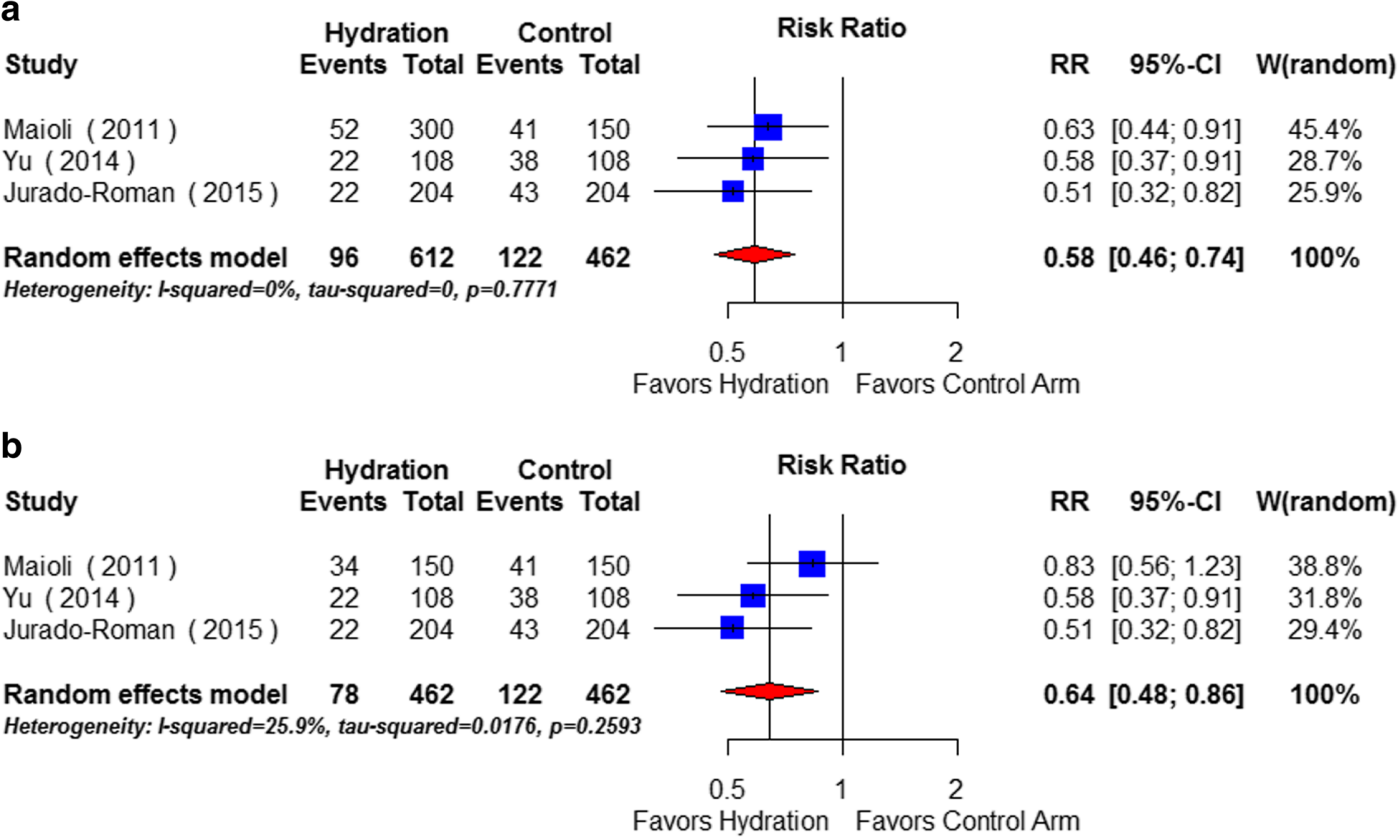
**a**. Effects of intravenous hydration lowering on risk of contrast-induced nephropathy (Hydration vs. No hydration, using random effects model) The event rate in different study arms is presented alongside the computed risk ratio (95% confidence interval [CI] (lower and upper limit) with *p* value. Forest plot shows effect size (solid squares) with 95% CI (black line through the solid squares), in terms of risk ratio for individual studies and pooled risk ratio (open diamonds) for random effects model at the bottom. Studies favouring reduction of risk with isotonic hydration are on the left of the centre line, and studies favouring control arm are on right of the centre line. **b**. Effects of intravenous saline hydration lowering on risk of contrast-induced nephropathy (Saline vs. No hydration) The event rate in different study arms is presented alongside the computed risk ratio (95% confidence interval [CI] (lower and upper limit) with p value. Forest plot shows effect size (solid squares) with 95% CI (black line through the solid squares), in terms of risk ratio for individual studies and pooled risk ratio (open diamonds) for random effects model at the bottom. Studies favouring reduction of risk with normal saline hydration are on the left of the centre line, and studies favouring control arm are on right of the centre line

The incidence of CIN was reported in 1074 patients who had completed the studies and were included in the final analysis. Isotonic fluid hydration was given to 612 patients and 462 patients were in control group. The overall incidence of CIN in patients receiving intravenous isotonic fluid hydration was 15.7% (96/612) compared with 26.4% (122/462) in the control group. Compared with no hydration, intravenous hydration significantly reduced the incidence of CI-AKI by 42% (RR 0.58; 95% CI: 0.46 to 0.74, *p* < 0.001) using a random effects model (Fig. 3a), so did that with fixed effect model (Additional file 5: Figure S3). There was no significant heterogeneity among the studies (I^2^ = 0.0%, chi-square = 0.50, df = 2, *p* = 0.7771). The effect of intravenous fluid remained when the cohort that received bicarbonate solution was excluded (RR: 0.64 [95%CI: 0.50 to 0.82]) (Fig. 3b).

### Secondary outcomes

**Fig. 4:**
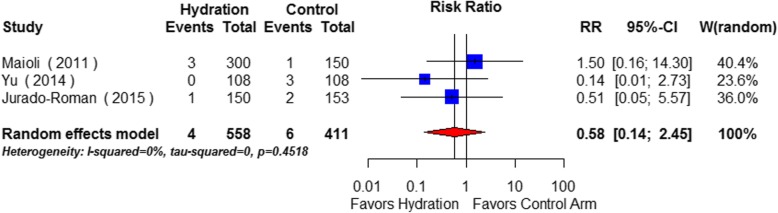
Effects of intravenous hydration lowering on risk of in-hospital requirement for dialysis (Hydration vs. No hydration)

**Fig. 5:**
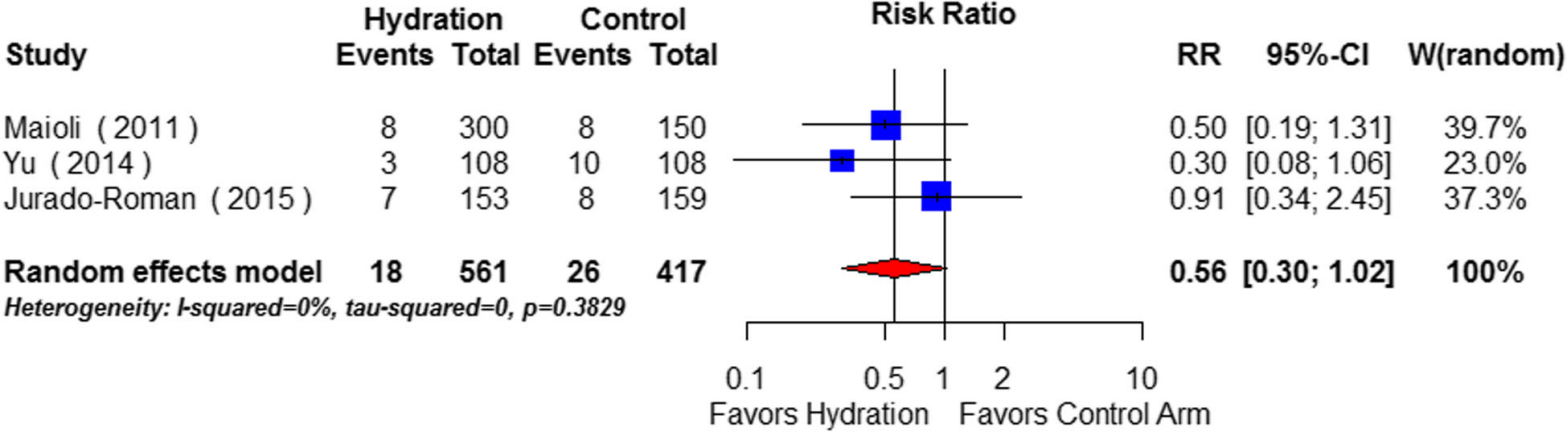
Effects of intravenous hydration lowering on risk of in-hospital all-cause mortality (Hydration vs. No hydration)

Overall 1.5% (15/969) of participants required dialysis and 4.5% (44/978) died in-hospital. There was no statistically significant reduction in dialysis requirement (RR 0.52, 95% CI 0.14–1.88) (Fig. 4) or all-cause In-hospital mortality (RR 0.56; 95% CI: 0.30 to 1.02) in the intravenous hydration group (Fig. 5).

The outcomes of heart failure and acute pulmonary edema, were not consistently reported. Luo et al. report that acute pulmonary edema developed in 13 (6%) patients (3 in the hydration group vs 10 in the no hydration group, *P* = 0.045), Jurado-Roman et al. report only that 42 patients (20.6%) in the hydration arm had their fluid ceased due to “heart failure despite reducing the rhythm of the infusion” (Table 1). Maioli et al. did not report heart failure or acute pulmonary edema.

### Trial sequential analysis

Assuming the modelled effect estimates, TSA estimated that an additional 790 study participants would need to be recruited beyond those studies to this point, to demonstrate a statistically significant improvement for In-hospital mortality. Similarly, an additional 8700 study participants would need to be studied to demonstrate an effect on the requirement for dialysis.

### Risk of bias across studies

The funnel plot showed minimal asymmetry and Egger’s regression intercept did not identify significant risk of bias (intercept = − 3.025 [95% CI: -18.545 to 12.495], df = 1, *p* = 0.244) (Additional file 6: Figure S4). However, this result should be interpreted with caution given that only 3 studies were included in the final analysis.

## Discussion

This study is, to our knowledge, the first systematic review and meta-analysis assessing the effect of hydration on prevention of CIN in patients with STEMI undergoing primary PCI. We demonstrated a statistically significant reduction in the incidence of CIN associated with isotonic hydration. The effects upon the requirement for dialysis and all-cause In-hospital mortality were not statistically significant, despite large estimated effect sizes. The interpretation of these results is tempered by the small number of clinically significant events, as well as the moderate quality of the included studies.

Besides the preventative effect of intravenous hydration on the risk CIN, the baseline patients characteristics, including renal insufficiency, age, heart failure were contribute to the development of CIN and even mortality [1, 14]. In addition, the association between CIN and mortality is strongly confounded by baseline clinical characteristics, large meta-analysis also showed CIN, as common complication of coronary angiography and/or coronary intervention (CAG/PCI), associated with increased lengthen of hospitalization, cardiovascular events, renal failure and mortality, so did that in Recent large STEMI Registry (e-PARIs) study among patients with STEMI undergoing primary PCI [15, 16]. Therefor the risk stratification of CIN was the key step for prevention for patients with STEMI, while the infarct-related artery (IRA) only PCI were not associated with lower risk of CIN, adequate hydration maybe the optimal prophylaxis for primary PCI [17]. In the present three included studies, there were no significant dereference in characteristics of patients and coronary procedure, including treated coronary, age, diabetes, female, renal and heart function, which were predictors for CIN and in-hospital [4, 5, 9].

This review confirms that CIN is common (20.3%) in patients with STEMI undergoing primary PCI and supports the use of hydration as prophylaxis in this setting. This is in contrast to a recent single centre study of high-risk patients receiving contrast during elective procedures where the incidence of CIN was only 2.7% [18]. Renal guidelines recommend the use of isotonic hydration, rather than no volume expansion, but the clinical setting of acute STEMI poses some challenges for such therapy [19, 20]. Avoidance of delays in reperfusion are paramount, and all three included studies commenced hydration with or after PCI, thus minimising delays in treatment. One study included a pre-hydration arm where there was no impact on door-to-reperfusion times [9].

A further priority is the avoidance of heart failure, which dramatically increases in-hospital mortality following STEMI [21]. Unfortunately the reporting of this outcome was not systematic, and the trials variably reported alterations to the fluid interventions in response to concerns about hydration state. Paradoxically, one study [Luo et al] reported significantly lower rates of acute pulmonary edema in the hydration arm (2.8% vs 9.3%). It is essential that the risk of heart failure is well understood before implementing routine hydration protocols for primary PCI.

Unfortunately, none of the included studies reported the delivered hydration volume in the control arm. It is likely that some hydration was administered as part of usual care, so it makes interpretation of the actual dose of fluid above and beyond usual care unclear. While the included studies did permit fluid for hypotensive patients, the possibility of systematic under-hydration in the control group cannot be excluded, which may have augmented the effect size seen in this analysis.

Potentially mitigating the concern around heart failure is the large, but not significant, effect sizes seen for the mortality and requirement for dialysis outcomes. While such effects are biologically plausible given the marked downstream impacts of acute kidney injury [22], the moderate study quality, the large sizes of the estimated effects, the small absolute number of events and the results of the TSA mean that more data is needed to define any effects. This might pose some challenges, as the treatment intervention is widely supported by guidelines and clinical practice, so cogent arguments would need to be made to ethically support withholding such treatment in a control arm of a study. Furthermore, the bearing of our findings upon future research and clinical practice rests heavily upon the weight given to the primary outcome of a 25% increase in serum creatinine. Others [23] have highlighted the questionable impact of small changes in serum creatinine upon clinically signficant outcomes, so we would argue that further studies in the setting of STEMI are needed.

The results of TSA indicate the challenges and opportunities facing future researchers. Although we demonstrated a consistent effect of hydration on CIN, given the methodological issues affecting the included studies the true effect size may be smaller than our results suggest. Similarly, the additional number of trial participants required to show an effect of hydration on important clinical endpoints, such as mortality and dialysis, may be significantly larger than the estimated 790 and 8700 from our analysis.

### Limitations

All trials were limited by being single centre, unblinded and without clearly defined allocation concealment mechanisms. Furthermore there was inconsistent reporting of cross-over between allocated groups and the volume of fluid received by the control arm was not reported in any study. It is also not possible to define the most effective hydration protocol as all three studies used different starting times, rates and durations of hydration. Further information is expected from the ATTEMPT study (NCT02067195) evaluating the efficacy of aggressive hydration volume compared with general hydration (≤500 mL) for CIN following primary PCI [24]. Finally, the relative benefit of sodium bicarbonate solutions versus sodium chloride cannot be addressed by this review and we are unable to comment on the utility of adjunctive therapies such as N-acetylcysteine or ascorbic acid.

## Conclusions

In this analysis of three single centre trials of moderate quality, we demonstrated a significant reduction in CIN after STEMI with intravenous hydration. The effects of hydration upon the important clinical end points of the need for acute dialysis and mortality were not signficant, but suggested large possible effects. Despite the widespread recommendations to use saline hydration across medicine, our findings support the need for further studies in the STEMI setting, especially focussed upon clarifying the appropriate volume of treatment as well as heart failure and In-hospital mortality effects.

## Additional files

Additional file 1: Search Strategy (DOCX 34 kb)

Additional file 2: **Table S1.** Additional characteristic of studies (DOCX 17 kb)

Additional file 3: **Figure S1.** Risk of bias summary: each risk of bias item for each included study (TIF 187 kb)

Additional file 4: **Figure S2.** GRADE assessment (TIF 42 kb)

Additional file 5: **Figure S3.** Effects of intravenous hydration lowering on risk of contrast-induced nephropathy (Hydration vs. No hydration, using fixed effects model) (TIF 12 kb)

Additional file 6: **Figure S4.** Funnel Plot for Subjective Assessment of Bias Among the Included Studies. Studies with larger sample size tend to accumulate at the top of the funnel and close to the centre line, with small studies toward the base. Funnel plot appears to have minimal asymmetry (TIFF 1456 kb)

## Abbreviations

CI: Confidence interval
CIN: Contrast-induced nephropathy
PCI: Percutaneous coronary intervention
RCT: Randomized controlled trial
ROS: Reactive oxygen species
RR: Risk ratio
SCr: Serum creatinine
STEMI: ST-segment elevation-myocardial infarction
TSA: Trial sequential analysis
