# Supplementary material for: Effects of intravenous hydration on risk of contrast induced nephropathy and in-hospital mortality in STEMI patients undergoing primary percutaneous coronary intervention: a systematic review and meta-analysis of randomized controlled trials
Source: BMC Cardiovasc Disord. 2019 Apr 8;19:87. doi: 10.1186/s12872-019-1054-y (PMC6454772; doi:10.1186/s12872-019-1054-y)
Supplement: Supplementary file 1 — Search Strategy (DOCX 34 kb) [file 12872_2019_1054_MOESM1_ESM.docx]

**Search strategy**

We searched MEDLINE, Embase, and Cochrane Central databases (from the date of inception until November 2018) to identify relevant articles. The searches performed in MEDLINE, Embase and Cochrane Central was essentially similar, except for the subject headings (see below).

1, Search strategy for MEDLINE

| Ovid MEDLINE(R) <1946 to September Week 3 2018> | | | |
| --- | --- | --- | --- |
| **#** | **Retrieval content** | **Results** | **Comments** |
| 1 | exp Contrast Media/ | 111190 |  |
| 2 | (contrast media or contrast medium or contrast material$ or contrast agent$ or contrast dye or radiographic contrast).tw. | 48590 |  |
| 3 | (radiocontrast media or radiocontrast medium or radiocontrast agent$).tw. | 519 |  |
| 4 | 1 or 2 or 3 | 128279 |  |
| 5 | (nephritis or nephropath$ or nephrotoxic$).tw. | 81955 |  |
| 6 | ((impair$ or damag$ or reduc$ or injur$ dysfunction$ or failure) adj2 (renal or kidney)).tw. | 117093 |  |
| 7 | exp Kidney Diseases/ | 479734 |  |
| 8 | exp nephritis/ or diabetic nephropathies/ | 92790 |  |
| 9 | exp renal insufficiency/ | 156586 |  |
| 10 | 5 or 6 or 7 or 8 or 9 | 541020 |  |
| 11 | 4 and 10 | 9871 |  |
| 12 | (contrast adj4 nephropathy).tw. | 1966 |  |
| 13 | (radiocontrast adj4 nephropathy).tw. | 213 |  |
| 14 | Kidney Diseases/ci [Chemically Induced] | 10624 |  |
| 15 | 11 or 12 or 13 or 14 | 19520 |  |
| 16 | exp Myocardial Infarction/ | 165296 |  |
| 17 | exp Coronary Thrombosis/ or coronary thrombosis.mp. | 8400 |  |
| 18 | acute coronary.mp. | 30092 |  |
| 19 | exp Angina, Unstable/ | 10701 |  |
| 20 | Myocardial infarct$.mp. | 215139 |  |
| 21 | heart infarct.mp. | 507 |  |
| 22 | acs.mp. | 15461 |  |
| 23 | ami.mp. | 15613 |  |
| 24 | (coronary adj3 syndrome$).mp. | 27947 |  |
| 25 | acute angina.mp. | 65 |  |
| 26 | (unstable adj3 angina).mp. | 15302 |  |
| 27 | unstable coronary.mp. | 770 |  |
| 28 | 16 or 17 or 18 or 19 or 20 or 21 or 22 or 23 or 24 or 25 or 26 or 27 | 254139 |  |
| 29 | hydration.mp. or exp hydration/ | 25332 |  |
| 30 | hydra*.tw. | 79990 |  |
| 31 | saline.mp. or exp sodium chloride/ | 201041 |  |
| 32 | exp bicarbonate/ or bicarbonate.mp. | 28123 |  |
| 33 | fluid.mp. or exp liquid/ | 435903 |  |
| 34 | 29 or 30 or 31 or 32 or 33 | 714215 |  |
| 35 | 15 and 28 and 34 | 41 |  |
| 36 | randomized controlled trial/exp or randomized controlled trial.tw. | 50227 |  |
| 37 | exp Random Allocation/ | 95920 |  |
| 38 | exp clinical trial/ | 807038 |  |
| 39 | exp single blind method/ | 25685 |  |
| 40 | exp double blind method/ | 147532 |  |
| 41 | (random$ adj5 trial$).tw. | 276327 |  |
| 42 | (random$ adj5 allocation$).tw. | 3227 |  |
| 43 | (blind$ adj5 method$).tw. | 17546 |  |
| 44 | 36 or 37 or 38 or 39 or 40 or 41 or 42 or 43 | 1024601 |  |
| 45 | 35 and 44 | 26 |  |
| 46 | remove duplicates from 45 | 26 |  |

2, Search strategy for Embase

| Embase <1974 to 2018 September 01> | | | |
| --- | --- | --- | --- |
| **#** | **Retrieval content** | **Results** | **Comments** |
| 1 | exp Contrast Media/ | 147387 |  |
| 2 | (contrast media or contrast medium or contrast material$ or contrast agent$ or contrast dye or radiographic contrast).tw. | 65506 |  |
| 3 | (radiocontrast media or radiocontrast medium or radiocontrast agent$).tw. | 746 |  |
| 4 | 1 or 2 or 3 | 166812 |  |
| 5 | (nephritis or nephropath$ or nephrotoxic$).tw. | 116992 |  |
| 6 | ((impair$ or damag$ or reduc$ or injur$ dysfunction$ or failure) adj2 (renal or kidney)).tw. | 178938 |  |
| 7 | exp Kidney Diseases/ | 808590 |  |
| 8 | exp nephritis/ or diabetic nephropathies/ | 109815 |  |
| 9 | exp renal insufficiency/ | 308963 |  |
| 10 | 5 or 6 or 7 or 8 or 9 | 858570 |  |
| 11 | 4 and 10 | 16309 |  |
| 12 | (contrast adj4 nephropathy).tw. | 3804 |  |
| 13 | (radiocontrast adj4 nephropathy).tw. | 256 |  |
| 14 | Kidney Diseases/ci [Chemically Induced] | 0 |  |
| 15 | 11 or 12 or 13 or 14 | 17679 |  |
| 16 | exp Myocardial Infarction/ | 340206 |  |
| 17 | exp Coronary Thrombosis/ or coronary thrombosis.mp. | 7807 |  |
| 18 | acute coronary.mp. | 67912 |  |
| 19 | exp Angina, Unstable/ | 22145 |  |
| 20 | Myocardial infarct$.mp. | 266204 |  |
| 21 | heart infarct.mp. | 261 |  |
| 22 | acs.mp. | 35235 |  |
| 23 | ami.mp. | 28837 |  |
| 24 | (coronary adj3 syndrome$).mp. | 65358 |  |
| 25 | acute angina.mp. | 86 |  |
| 26 | (unstable adj3 angina).mp. | 27493 |  |
| 27 | unstable coronary.mp. | 1116 |  |
| 28 | 16 or 17 or 18 or 19 or 20 or 21 or 22 or 23 or 24 or 25 or 26 or 27 | 440718 |  |
| 29 | hydration.mp. or exp hydration/ | 46103 |  |
| 30 | hydra*.tw. | 111953 |  |
| 31 | saline.mp. or exp sodium chloride/ | 298320 |  |
| 32 | exp bicarbonate/ or bicarbonate.mp. | 59758 |  |
| 33 | fluid.mp. or exp liquid/ | 672182 |  |
| 34 | 29 or 30 or 31 or 32 or 33 | 1094819 |  |
| 35 | 15 and 28 and 34 | 253 |  |
| 36 | randomized controlled trial/exp or randomized controlled trial.tw. | 77969 |  |
| 37 | exp Random Allocation/ | 79625 |  |
| 38 | exp clinical trial/ | 1328502 |  |
| 39 | exp single blind method/ | 32408 |  |
| 40 | exp double blind method/ | 152952 |  |
| 41 | (random$ adj5 trial$).tw. | 437871 |  |
| 42 | (random$ adj5 allocation$).tw. | 4906 |  |
| 43 | (blind$ adj5 method$).tw. | 33370 |  |
| 44 | 36 or 37 or 38 or 39 or 40 or 41 or 42 or 43 | 1595552 |  |
| 45 | 35 and 44 | 103 |  |
| 46 | remove duplicates from 45 | 102 |  |

3, Cochrane Central

| EBM Reviews - Cochrane Central Register of Controlled Trials < September 2018> | | | |
| --- | --- | --- | --- |
| **#** | **Retrieval content** | **Results** | **Comments** |
| 1 | exp Contrast Media/ | 3625 |  |
| 2 | (contrast media or contrast medium or contrast material$ or contrast agent$ or contrast dye or radiographic contrast).tw. | 3420 |  |
| 3 | (radiocontrast media or radiocontrast medium or radiocontrast agent$).tw. | 56 |  |
| 4 | 1 or 2 or 3 | 5365 |  |
| 5 | (nephritis or nephropath$ or nephrotoxic$).tw. | 7098 |  |
| 6 | ((impair$ or damag$ or reduc$ or injur$ dysfunction$ or failure) adj2 (renal or kidney)).tw. | 9142 |  |
| 7 | exp Kidney Diseases/ | 13610 |  |
| 8 | exp nephritis/ or diabetic nephropathies/ | 2315 |  |
| 9 | exp renal insufficiency/ | 7443 |  |
| 10 | 5 or 6 or 7 or 8 or 9 | 24130 |  |
| 11 | 4 and 10 | 838 |  |
| 12 | (contrast adj4 nephropathy).tw. | 694 |  |
| 13 | (radiocontrast adj4 nephropathy).tw. | 51 |  |
| 14 | Kidney Diseases/ci [Chemically Induced] | 16 |  |
| 15 | 11 or 12 or 13 or 14 | 1093 |  |
| 16 | exp Myocardial Infarction/ | 9797 |  |
| 17 | exp Coronary Thrombosis/ or coronary thrombosis.mp. | 477 |  |
| 18 | acute coronary.mp. | 5330 |  |
| 19 | exp Angina, Unstable/ | 1032 |  |
| 20 | Myocardial infarct$.mp. | 23428 |  |
| 21 | heart infarct.mp. | 16 |  |
| 22 | acs.mp. | 2794 |  |
| 23 | ami.mp. | 2615 |  |
| 24 | (coronary adj3 syndrome$).mp. | 5204 |  |
| 25 | acute angina.mp. | 8 |  |
| 26 | (unstable adj3 angina).mp. | 2873 |  |
| 27 | unstable coronary.mp. | 187 |  |
| 28 | 16 or 17 or 18 or 19 or 20 or 21 or 22 or 23 or 24 or 25 or 26 or 27 | 28158 |  |
| 29 | hydration.mp. or exp hydration/ | 2386 |  |
| 30 | hydra*.tw. | 3593 |  |
| 31 | saline.mp. or exp sodium chloride/ | 25236 |  |
| 32 | exp bicarbonate/ or bicarbonate.mp. | 2772 |  |
| 33 | fluid.mp. or exp liquid/ | 20741 |  |
| 34 | 29 or 30 or 31 or 32 or 33 | 48885 |  |
| 35 | 15 and 28 and 34 | 59 |  |
| 36 | randomized controlled trial/exp or randomized controlled trial.tw. | 82842 |  |
| 37 | exp Random Allocation/ | 20614 |  |
| 38 | exp clinical trial/ | 173 |  |
| 39 | exp single blind method/ | 18317 |  |
| 40 | exp double blind method/ | 127938 |  |
| 41 | (random$ adj5 trial$).tw. | 301181 |  |
| 42 | (random$ adj5 allocation$).tw. | 4585 |  |
| 43 | (blind$ adj5 method$).tw. | 41238 |  |
| 44 | 36 or 37 or 38 or 39 or 40 or 41 or 42 or 43 | 425890 |  |
| 45 | 35 and 44 | 32 |  |
| 46 | remove duplicates from 45 | 32 |  |
