# Supplementary material for: Effects of intravenous hydration on risk of contrast induced nephropathy and in-hospital mortality in STEMI patients undergoing primary percutaneous coronary intervention: a systematic review and meta-analysis of randomized controlled trials
Source: BMC Cardiovasc Disord. 2019 Apr 8;19:87. doi: 10.1186/s12872-019-1054-y (PMC6454772; doi:10.1186/s12872-019-1054-y)
Supplement: Supplementary file 2 — Table S1. Additional characteristic of studies (DOCX 17 kb) [file 12872_2019_1054_MOESM2_ESM.docx]

| **Study** | **Mean duration of following the Screatinine after primary PCI** | **Baseline Screatinine (mg/dL)**  **(Intervention vs Control)** | **Baseline eGFR (mL/min/1.73m^2^ )**  **(Intervention vs Control)** | **Baseline LVEF (%) (Intervention vs Control)** | **Age (yrs)**  **(Intervention vs Control)** | **Female sex**  **(Intervention vs Control)** | **Diabetes**  **(Intervention vs Control)** | **Killip class > 1**  **(intervention vs control)，**  **Cardiogenic shock**  **(Intervention vs Control)** | **Rate of Stenting，**  **Post-procedural TIMI flow 3**  **(Intervention vs Control)** | **Culprit lesion:LAD**  **(Intervention vs Control)** | **Hydration**  **(Intervention vs Control)** |
| --- | --- | --- | --- | --- | --- | --- | --- | --- | --- | --- | --- |
| Maioli et.al.  (2011) | Within 3 days after administration of the contrast medium | 1.10±0.4^※^ and 1.09±0.3^#^ vs 1.08±0.3,  P=0.41 (among the 3 groups) | 74±22^※^ and 75±23^#^ vs 78±20,  P=0.25 (among the 3 groups) | 43 ± 7^※^ and 43 ± 8^#^ vs 42 ± 9, P=0.67 (among the 3 groups) | 66±12^※^ and 65±13^#^ vs 64±12,  P=0.38 (among the 3 groups) | 27.3% (41/150)^※^  and 23.3 (35/150) ^#^ vs  26.6% (40/150),  P=0.79 (among the 3 groups) | 20.7% (31/150) ^※^ and 20.7 (31/150) ^#^ vs 22.7% (34/150),  P=0.67 (among the 3 groups) | 20.0% (30/150)^※^  and 22.7% (34/150) ^#^ vs  25.3% (38/150),  P=0.54 (among the 3 groups);  6.0% (9/150) and 4.0% (6/150/ vs 5.3% (8/150),  P=0.60 (among the 3 groups) | 92% (92/150) ^※^ and 90.6% (136/150) ^#^ vs 89.3% (134/150),  P=0.88 (among the 3 groups);  Unknown | Unknown | Mean volume: 1021±196ml vs Unknown |
| Luo et.al.  (2014) | Within 72 hs after administration of the contrast medium | 0.86±0.15 vs 0.88±0.14,  P=0.27 | 71.7±22.6 vs 69.4±23.2,  P=0.35 | 46 ± 13 vs 48 ± 10, P=0.205 | 67.2(58-74) vs 66.7(57-75),  P=0.76 | 30.6% (33/108) vs 38% (31/108), | 25.9% (28/108) vs 24.1% (26/108), P=0.31 | 37.1% (41/108) vs 34.3% (37/108);  Unknown | 91.7% (99/108) vs 90.7% (98/108), P=0.81;  93.5% (101/108) vs 94.4% (102/108), P=0.78 | 57.4% (62/108) vs 54.6 (59/108), P=0.68 | Mean rate: 0.75ml/kg/hour vs Unknown |
| Jurado-Román et.al.  (2015) | Within 3 days after administration of the contrast medium | Unknown | 90±21 vs 88±54,  P=0.45 | 48 ± 6 vs 51 ± 9, P=0.85 | 61.7±14 vs 63.9±12,  P=0.25 | 27.9% (24/204) vs 25.5% (52/204) | 23.5% (48/204) vs 21.6% (44/204), P=0.61 | 13.2% (27/204) vs 16.1% (33/204),  P=0.65;  Unknown | Unknown,  Unknown | Unknown | Mean volume: 1720±234 ml vs Unknown |
|  |  |  |  |  |  |  |  |  |  |  |  |

PCI=Percutaneous Coronary Intervention; GFR=Glomerular filtration rate; LVEF= left ventricular ejection fration; TIMI= Thrombolysis in Myocardial Infarction; LAD= Left anterior descending artery.

^※^late hydration group: 3 mL/kg of sodium bicarbonate solution in 1 hour, starting in the emergency room, followed by infusion of 1 mL/kg per hour for 12 hours after PCI; ^#^early hydration group: received isotonic saline (1 mL/kg per hour 0.9% sodium chloride) for 12 hours immediately after PCI.
