# Supplementary figures and images for: Effects of intravenous hydration on risk of contrast induced nephropathy and in-hospital mortality in STEMI patients undergoing primary percutaneous coronary intervention: a systematic review and meta-analysis of randomized controlled trials
Source: BMC Cardiovasc Disord. 2019 Apr 8;19:87. doi: 10.1186/s12872-019-1054-y (PMC6454772; doi:10.1186/s12872-019-1054-y)

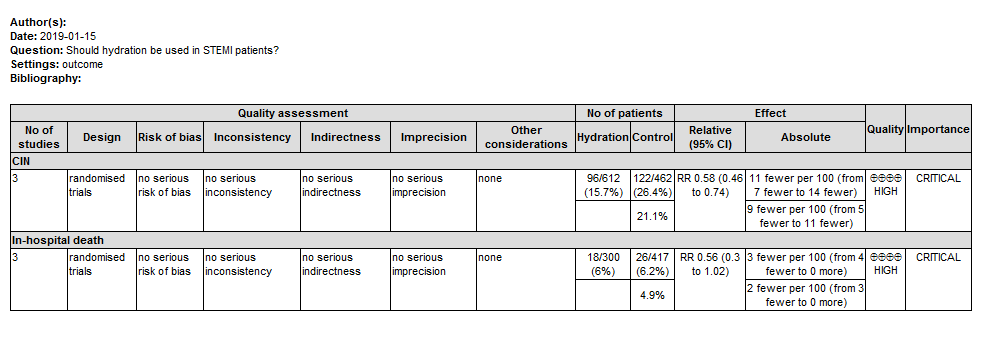

Supplement: Supplementary file 4 — Figure S2. GRADE assessment (TIF 42 kb) [file 12872_2019_1054_MOESM4_ESM.tif]

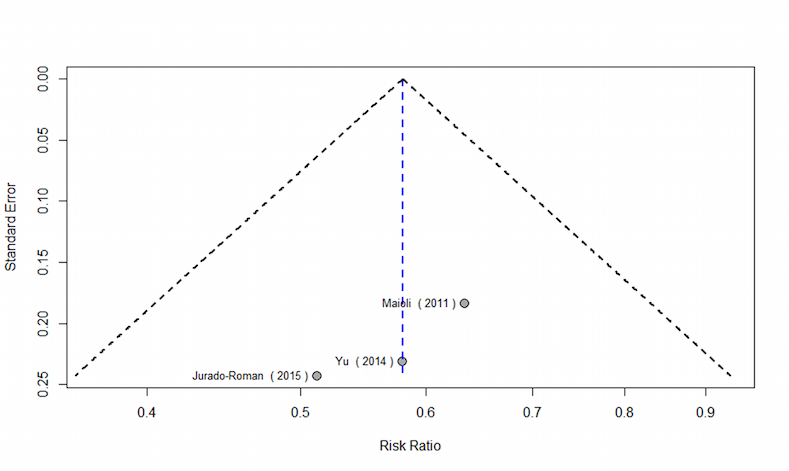

Supplement: Supplementary file 6 — Figure S4. Funnel Plot for Subjective Assessment of Bias Among the Included Studies. Studies with larger sample size tend to accumulate at the top of the funnel and close to the centre line, with small studies toward the base. Funnel plot appears to have minimal asymmetry (TIFF 1456 kb) [file 12872_2019_1054_MOESM6_ESM.tiff]
